# Case Report: A diagnostic challenge: rare adverse effects of GnRH analogue therapy in a late pubertal pediatric patient

**DOI:** 10.3389/fped.2026.1850033

**Published:** 2026-07-17

**Authors:** Dao-Chen Lin, Pei-Shan Tsai, Tao-Yeuan Wang, Wen-Ko Su

**Affiliations:** 1 Department of Radiology, Taipei Veterans General Hospital, Taipei City, Taiwan; 2 Division of Endocrine and Metabolism, Department of Medicine, Taipei Veterans General Hospital, Taipei City, Taiwan; 3 School of Medicine, College of Medicine, National Yang Ming Chiao Tung University, Taipei City, Taiwan; 4 Department of Radiology, MacKay Memorial Hospital, Taipei, Taiwan; 5 Department of Medicine, Mackay Medical College, New Taipei City, Taiwan; 6 Mackay Junior College of Medicine, Nursing and Management, New Taipei City, Taiwan; 7 Department of Pathology, MacKay Memorial Hospital, Taipei, Taiwan; 8 Department of Radiology, Mackay Memorial Hospital, Hsinchu Branch, Hsinchu City, Taiwan

**Keywords:** downregulation, flare-up, gonadotropin-releasing hormone (GnRH), ovary torsion, surgery

## Abstract

We present the case of a 12-year-old post-menarchal female (bone age: 13 years; 30th percentile for height) who received an initial gonadotropin-releasing hormone (GnRH) analogue injection for height augmentation to maximize adult height potential prior to imminent epiphyseal fusion. Immediately following the injection, she developed acute-onset abdominal pain, emesis, and diarrhea. Refractory to conservative management, her clinical status prompted pelvic ultrasonography and subsequent contrast-enhanced CT imaging, which revealed significant bilateral ovarian enlargement, multiple cystic masses, and congestion of the left vascular pedicle highly indicative of adnexal torsion. Urgent surgical exploration confirmed bilateral ovarian cyst formation and left-sided ovarian torsion. Following an unremarkable post-operative recovery, GnRH analogue therapy was permanently discontinued. This case underscores the critical need to recognize the heightened risk of rapid ovarian hyperstimulation and subsequent torsion when initiating these medications in post-menarchal or late pubertal females, in whom mature follicles are already present. While GnRH analogues generally maintain a favorable safety profile, clinician vigilance regarding this severe ovarian complication in older pediatric cohorts is vital to prevent diagnostic delays and ensure prompt surgical intervention.

## Introduction

Ovarian torsion is the complete or partial twisting of the vascular pedicle within the suspensory ligament. This mechanical compromise leads to lymphatic obstruction, followed by venous congestion and, ultimately, arterial occlusion. Adnexal torsion in pediatric and adolescent populations accounts for approximately 15% of all torsion cases (1). While it can occur at any age—from infancy through 18 years—up to 52% of pediatric cases occur between the ages of 9 and 14, with a median age of 11 years (1, 2).

More than half of pediatric adnexal torsion cases are secondary to underlying adnexal pathology. These include cystic teratomas or dermoid cysts (31%), follicular or hemorrhagic ovarian cysts (23%–33%), and, less frequently, paraovarian/paratubal cysts, cystadenomas, or hydrosalpinx (3, 4). As a critical surgical emergency, adnexal torsion is ranked as the fifth most common gynecologic emergency in patients under 20 years of age (5–8). Without urgent surgical intervention, the ovary may undergo necrosis, leading to long-term consequences for future fertility. Consequently, prompt diagnosis and management are vital.

Unlike the physiological, rhythmic pulses of endogenous gonadotropin-releasing hormone (GnRH), these synthetic analogues maintain a constant level of activity. This continuous exposure leads to a paradoxical desensitization of the pituitary gland, effectively downregulating the HPG axis and halting pubertal progression.

We herein present the case of a late-pubertal 12-year-old girl receiving GnRH analogue therapy. Shortly after the first administration of the medication, she developed left ovarian torsion—a rare but clinically significant complication potentially associated with the initiation of GnRH analogue therapy.

## Case report

The patient, a 12-year-old girl with an unremarkable medical history, had been followed regularly at the pediatric endocrinology clinic for short stature since the age of 10. At her evaluation at age 12, her height was 143.2 cm (30th percentile), her body weight was 34.7 kg, and her bone age was determined to be 13 years. Physical examination revealed advanced pubertal development, characterized by Tanner breast stage 4 and pubic hair stage 2. She had also recently experienced menarche. The baseline serum estradiol level was 32.56 pg/mL. Following extensive counseling with her family regarding height augmentation strategies, a clinical decision was made to initiate off-label therapy to delay epiphyseal fusion. Subsequently, she received her first intramuscular dose of a 3-month depot formulation of triptorelin pamoate (Diphereline®).

One week after the injection, she developed acute abdominal pain localized primarily in the epigastric area, accompanied by nausea, vomiting, and diarrhea. She was subsequently brought to emergency department (ED). Upon arrival, her vital signs were relatively stable: body temperature 37.4 °C, pulse rate 68 beats/min, respiratory rate 24 breaths/min, and blood pressure 100/51 mmHg. Laboratory results revealed leukocytosis, and an abdominal x-ray showed a dilated bowel gas pattern.

Due to a lack of bowel movements and persistent pain, an abdominal sonogram was performed. The scan revealed two large multicystic pelvic masses (the upper measuring 11.5 × 5.8 cm and the lower 7.2 × 6.7 cm) alongside moderate ascites (Figures 1A,B). A computed tomography (CT) scan with and without contrast (Figure 2) further demonstrated bilateral ovarian enlargement with a multicystic appearance and suspected engorgement of the vascular pedicles.

**Figure 1:**
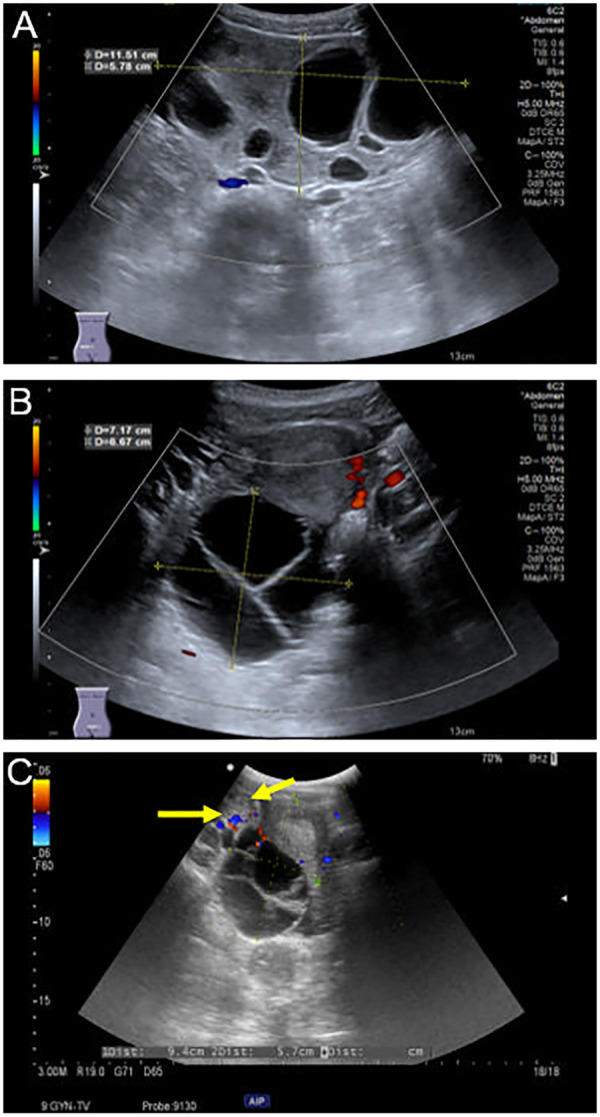
Ultrasonographic evidence of bilateral ovarian enlargement. **(A)** Gray-scale sonography demonstrates a markedly enlarged left ovary with a multiloculated cystic architecture, the maximal diameter measuring approximately 11.5 cm. **(B)** The right ovary is likewise enlarged, exhibiting a similar multicystic pattern with a greatest dimension of roughly 7.2 cm. **(C)** Color Doppler follow-up shows persistent enlargement of the left ovary with multiple peripheral cysts and an engorged vascular pedicle displaying the classic “whirlpool” sign (arrows), findings characteristic of ovarian torsion.

**Figure 2:**
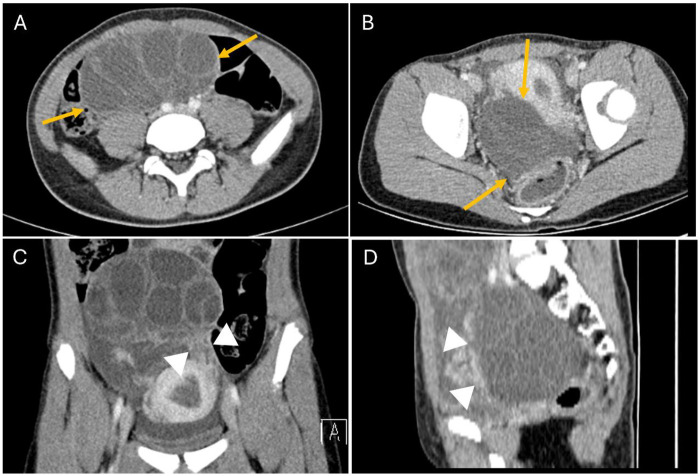
Cross-sectional computed tomotraphy illustrating bilateral ovarian enlargement and pedicle thickening. **(A,C)** Axial and coronal CT images demonstrate a markedly enlarged left ovary situated along the midline superior to the uterus. The ovary exhibits a multiloculated cystic morphology (arrows) and an accompanying thickened vascular pedicle (arrowheads). **(B,D)** Axial and oblique-coronal CT images reveal an enlarged right ovary within the cul-de-sac, likewise displaying a multicystic architecture (arrows) and a prominently thickened pedicle (arrowheads).

Although the patient's pain improved slightly after a bowel movement, she was admitted for further management under a tentative diagnosis of bilateral adnexal masses and suspected ovarian torsion. Pediatric oncology, endocrinology, and surgical teams were consulted to evaluate the possibility of ovarian tumors coexisting with torsion. A follow-up abdominal sonogram confirmed the absence of blood flow to the left ovary (Figure 1C). Subsequent laboratory testing revealed a profoundly elevated serum estradiol level of 6,089.98 pg/mL.

The patient underwent an exploratory laparotomy. Intraoperative findings confirmed torsion of the left ovary and fallopian tube with gangrenous changes. The right ovary was also enlarged and multi-cystic but showed no signs of ischemia or gangrene (Figure 3). A frozen section biopsy of the left ovarian mass identified follicular cysts with no evidence of malignancy. Consequently, a left salpingo-oophorectomy and fluid cytology of the right ovary were performed successfully. Postoperative blood tests revealed estradiol level <10 pg/mL. Final pathology confirmed left ovarian cystic follicular lesions associated with torsion (Figure 4). The patient recovered well, was discharged, and continues to be monitored in our outpatient department. At a later follow-up, the patient's serum estradiol level was 51.58 pg/mL.

**Figure 3:**
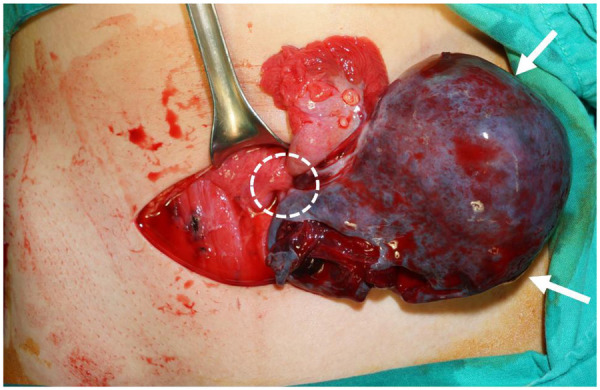
Intra-operative gross appearance of the left adnexa. The left ovary is markedly enlarged and exhibits extensive hemorrhagic infarction (arrows). A 270-degree torsion of the distal fallopian tube is evident (encircled), accounting for the vascular compromise.

**Figure 4:**
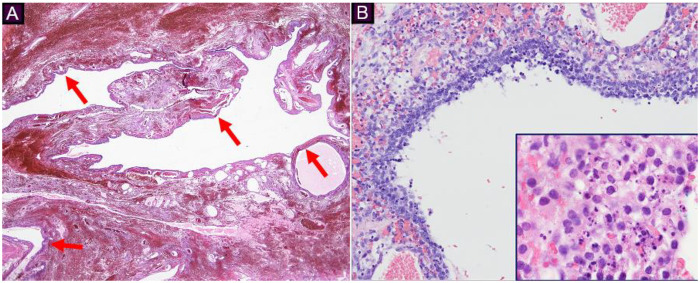
Histopathological evaluation of hemorrhagic ovarian torsion secondary to follicular cyst. **(A)** Low-power photomicrograph (H&E stain, 10x) demonstrating multiple distended cystic follicular lesions (indicated by red arrows). The surrounding ovarian stroma exhibits extensive interstitial hemorrhage and congestive necrosis, pathognomonic for acute adnexal torsion. **(B)** Intermediate-power magnification (H&E stain, 200x) of a follicular cyst wall, characterized by a preserved granulosa cell layer. Insert: High-magnification view (H&E stain, 1000x) revealing cellular details of the granulosa lining with prominent apoptotic bodies and nuclear pyknosis, reflecting acute ischemic injury following the torsional event.

## Discussion

Adnexal torsion is a critical gynecological emergency, recognized as the fifth most frequent cause of surgical emergencies in pediatric and adolescent populations. It carries a reported prevalence of 2.7% and an incidence rate of 4.9 per 100,000 among females under the age of 20 (5–8), accounting for approximately 15% of all torsion cases within this demographic (1).

While adnexal torsion can occur at any pediatric age from infancy to 18 years, approximately 52% of cases cluster between the ages of 9 and 14, with a median age of onset at 11 years (1, 2). Although up to 25% of pediatric cases involve morphologically normal ovaries, the vast majority (51%–84%) are associated with underlying adnexal pathology. The most prevalent etiologies include cystic teratomas or dermoid cysts (∼31%), followed closely by follicular or hemorrhagic ovarian cysts (23%–33%); less frequent causes include paraovarian or paratubal cysts, cystadenomas, and hydrosalpinx (3, 4). Additionally, conditions that induce ovarian enlargement, such as Polycystic Ovary Syndrome (PCOS), increase both ovarian volume and weight, creating significant predisposing factors for torsion. Mechanistically, the pathophysiologic cascade of torsion involves initial compression of venous and lymphatic outflow within the infundibulopelvic pedicle, followed by arterial obstruction, ultimately culminating in ovarian ischemia, hemorrhagic infarction, and necrosis (9–11).

In the field of reproductive endocrinology, a low-dose GnRH agonist is frequently utilized as an ovulation trigger specifically because its stimulatory flare effect is short-lived compared to human chorionic gonadotropin (hCG) (12). However, a critical distinction must be made regarding the dosing mechanics in pediatric care: the depot formulation administered for pubertal suppression or adult height augmentation contains a significantly higher dose designed for long-term receptor desensitization and down-regulation (13, 14). When introduced to an already mature ovarian microenvironment, this massive initial depot release can trigger an uncontrolled, exaggerated gonadotropin surge. This unique pathophysiological cascade can lead to rapid follicular hyperstimulation and massive cyst formation, mimicking a severe form of ovarian hyperstimulation syndrome complicated by adnexal torsion. Similar rare occurrences of massive ovarian cyst formation during the initial flare phase of high-dose GnRH agonist protocols have been sporadically reported in adult controlled ovarian stimulation (15), but its manifestation during pediatric off-label growth therapy remains exceptionally rare.

In the present case, the patient developed bilateral ovarian enlargement with multiple follicular cysts shortly after receiving her first 3-monthly depot of triptorelin. The hypothesized mechanism involves this initial, exaggerated “flare” effect—an acute surge of gonadotropin release that hyperstimulates the ovaries and drives rapid follicle recruitment (16). Because this patient was nearing the completion of puberty (Tanner breast stage 4, bone age 13 years), her ovarian architecture already possessed a robust pool of gonadotropin-sensitive, advanced antral follicles. Consequently, her risk profile for ovarian cystogenesis mirrored that of a post-pubertal or adult female undergoing ovarian stimulation. Similar to the mechanics seen in PCOS, these acutely enlarged, asymmetric ovaries likely served as a mechanical fulcrum, predisposing the adnexa to axial rotation, compromising vascular supply, and leading to the ischemic necrosis confirmed during surgery and histopathological examination.

The tight temporal association between Diphereline® administration and the onset of acute symptoms, coupled with imaging and surgical findings, strongly supports a causal relationship between the initial GnRH agonist flare and rapid ovarian hyperstimulation. Although such events are rare, this case highlights the critical necessity of close clinical and ultrasonographic monitoring for ovarian hyperstimulation and related complications in adolescent patients initiating GnRH analogue therapy, particularly those with advanced baseline pubertal status. While histological findings confirmed a benign etiology, the resulting loss of the left ovary underscores the critical need for early recognition and prompt surgical intervention to preserve future reproductive and endocrine function.

## Conclusion

This case underscores a critical, population-specific vulnerability: the transient “flare-up” effect of initiating GnRH agonist (GnRHa) therapy can serve as a rare but significant trigger for rapid ovarian cyst formation and subsequent mechanical torsion. Crucially, this risk is heightened in post-menarchal or late-pubertal females due to the presence of mature, highly responsive ovarian follicles. Ultimately, this case serves as a cautionary example: the risk of using a depot GnRHa in a late-pubertal patient may outweigh the potential benefits of height augmentation. To optimize clinical outcomes and preserve long-term reproductive function in this demographic, early diagnostic recognition, prompt imaging, and emergent surgical intervention remain paramount.

## Data Availability

The original contributions presented in the study are included in the article/Supplementary Material, further inquiries can be directed to the corresponding author.
